# HSPs/STAT3 Interplay Sustains DDR and Promotes Cytokine Release by Primary Effusion Lymphoma Cells

**DOI:** 10.3390/ijms24043933

**Published:** 2023-02-15

**Authors:** Roberta Gonnella, Andrea Arena, Roberta Zarrella, Maria Saveria Gilardini Montani, Roberta Santarelli, Mara Cirone

**Affiliations:** Department of Experimental Medicine, Sapienza University of Rome, Viale Regina Elena 324, 00161 Rome, Italy

**Keywords:** PEL, HSP27, HSP70, HSP90, DDR, STAT3, cytokines

## Abstract

Primary effusion lymphoma (PEL) is a rare and aggressive B-cell lymphoma, against which current therapies usually fail. In the present study, we show that targeting HSPs, such as HSP27, HSP70 and HSP90, could be an efficient strategy to reduce PEL cell survival, as it induces strong DNA damage, which correlated with an impairment of DDR. Moreover, as HSP27, HSP70 and HSP90 cross talk with STAT3, their inhibition results in STAT3 de-phosphorylation and. On the other hand, the inhibition of STAT3 may downregulate these HSPs. These findings suggest that targeting HSPs has important implications in cancer therapy, as it can reduce the release of cytokines by PEL cells, which, besides affecting their own survival, could negatively influence anti-cancer immune response.

## 1. Introduction

Primary effusion lymphoma (PEL) is a highly aggressive B-cell lymphoma, associated with Kaposi-sarcoma-associated herpesvirus (KSHV), that poorly responds to anti-cancer therapies. Therefore, more investigations are needed to improve the treatment of patients affected by such malignancy. Previous studies performed in vitro and in vivo in other laboratories, alongside ours, have shown that targeting molecular pro-survival pathways, constitutively activated in this lymphoma, may represent a promising therapeutic option. Among those, the inhibition of STAT3, mTOR and NRF2, have demonstrated some success in impairing PEL cell survival [1,2,3,4]. Of note, most of these pathways also control the release of cytokines, molecules that contribute to PEL cell growth [5], beside dysregulating anti-cancer immune response. However, we have shown that despite exerting a cytotoxic effect against PEL, the inhibition of pro-survival pathways, such as STAT3, activated pro-survival processes, such as autophagy, as a compensatory mechanism to cope with the downregulation of heat shock proteins (HSPs) induced by this treatment [4].

HSPs, a family of proteins classified based on their molecular weight, are important pro-survival molecules, of which the inhibition represents a potential strategy to be used in anti-cancer therapy [6]. However, despite the efforts made, no HSP inhibitors have reached Food and Drug Administration (FDA) approval so far, suggesting that more studies need to be performed in this direction to obtain this goal. The use of HSP inhibitors can be particularly promising, as it may allow to selectively kill cancer cells while sparing normal cells [7], as the former express a higher level of HSPs, such as HSP27, HSP70 and HSP90, compared to the normal counterpart [8]. The different expression of HSPs is due to the necessity of the cancer cells to fold the high amount of proteins that they synthesize and face the stressful conditions in which they are forced to survive. Alongside assisting the folding and stabilization of nascent proteins, HSPs display several other functions that also depend on their subcellular localization [9]. Interestingly, heat shock transcription factor1 (HSF1), the main transcription factor devolved to HSP transcription, may represent another promising target in the treatment of cancer [10], including PEL [4]. HSF1, indeed, has multiple target genes involved in key cellular processes, including an unfolded protein response (UPR), autophagy and apoptosis [11]. We have previously shown that targeting HSP70 could selectively kill PEL cells, but not primary B lymphocytes that display a very low basal expression of this HSP. As an underlying mechanism, we found that HSP70 inhibition induced damage of the lysosomal membrane, leakage of the lysosomal enzymes into the cytoplasm and necroptotic cell death [7]. The inhibition of HSP90 has also been reported to be cytotoxic against PEL cells and induce apoptosis by inhibiting the transcriptional activity of the nuclear factor-kappa B (NF-κB) [12]. Regarding HSP27, as far as we know, no studies have investigated the possibility to target this small HSP in the treatment of PEL. Of note is that among the numerous proteins stabilized by HSPs, there are oncogenes such as mutp53 [13] and c-MYC [14], epidermal growth factor receptor (EGFR) and the molecules involved in its mediated signaling cascade [15], and proteins involved in processes such as cell cycle progression and angiogenesis [6].

Last, but not least, HSPs sustain the DNA damage response (DDR) [16], a process particularly important for the survival of cancer cells, in which DNA damage occurs also in basal conditions due to the high replication rate. Moreover, DNA damage may be exacerbated by treatments with DNA-damaging agents widely used to treat cancer. HSPs may stabilize proteins belonging to all DNA repair pathways, activated based on the type of DNA damage [16].

The DNA repair process indeed includes different pathways, such as the direct damage reversal, base excision repair (BER), nucleotide excision repair (NER), mismatch repair (MMR), homologous recombination (HR) and non-homologous end-joining (NHEJ). Based on this background, here we investigated the impact of HSP27, HSP70 and HSP90 inhibition on DNA damage and cell death in PEL cells, and correlate these effects with the possible downregulation of proteins involved in the DNA repair pathways. Given that HSPs may also act as chaperone-kinase-activating pathways, such STAT3 [17], we then evaluated whether HSP inhibition could affect this pathway constitutively activated in PEL cells and strongly involved in their survival. Moreover, we explored whether STAT3 inhibition could influence the expression levels of HSPs and also alter the DDR molecules in these cells. Finally, the possibility to sensitize PEL cells to the poly (ADP-ribose) polymerase (PARP) inhibitor, AZD2461, by pre-treating them with HSP or STAT3 inhibitors, was explored. Discovering the interconnections between pro-survival molecules, and interrupting them, could unveil new unexpected weakness points which could be exploited to potentiate the efficacy of pre-existing therapies.

## 2. Results

### 2.1. HSP27, HSP70 and HSP90 Inhibition Strongly Impairs PEL Cell Survival

**Figure 1:**
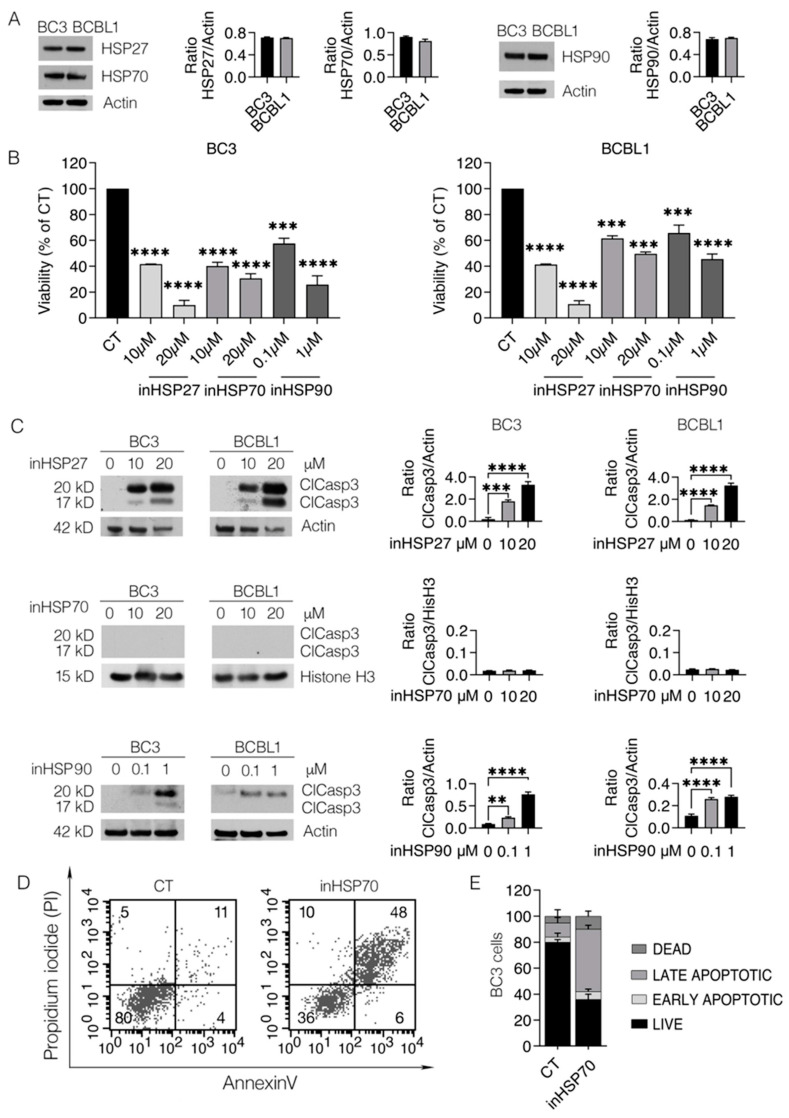
Inhibitors of HSPs strongly impair PEL survival. (**A**) Protein expression levels of HSP27, HSP70 and HSP90 were evaluated by Western blot analysis in BC3 and BCBL1. Actin was used as a loading control and one representative experiment is shown. The histograms represent the densitometric analysis of the ratio of specific protein/actin. Data are represented as the mean plus S.D. BC3 and BCBL1 cells were treated with inHSP27 (J2) (10–20 µM), inHSP70 (PES) (10–20 µM) or inHSP90 (17AAG) (0.1–1 µM) for 24 h and (**B**) cell survival was evaluated by a trypan blue exclusion assay. The histograms represent the mean of the percentage of cell viability relative to the control plus S.D. *p* value: *** <0.001; **** <0.0001. BC3 and BCBL1 cells were treated with inHSP27 (10–20 µM), inHSP70 (10–20 µM) or inHSP90 (0.1–1 µM) for 24 h and (**C**) protein expression levels of cleaved caspase-3 (ClCasp3) were evaluated by Western blot analysis. Actin or histone H3 was used as a loading control and one representative experiment is shown. The histograms represent the densitometric analysis of the ratio of specific protein/actin or histone H3. Data are represented as the mean plus S.D. *p* value: ** <0.01; *** <0.001; **** <0.0001. BC3 cells were treated with inHSP70 (20 µM) for 6 h, then double-stained with annexin V/PI and analyzed by FACS analysis. (**D**) One representative experiment for BC3 is shown. (**E**) The histograms represent the mean of percentage of live (annexin-V-negative, PI-negative), early apoptotic (annexin-V-positive, PI-negative), late apoptotic (annexin-V-positive, PI-positive) and dead cells (annexin-V-negative, PI-positive).

Similarly to the majority of cancer cells, BC3 and BCBL1 PEL cells express high levels of HSP27, 70 and 90 (Figure 1A). To evaluate the role of these HSPs in cell survival, PEL cells were treated with HSP27, HSP70 and HSP90 inhibitors, namely J2, PES and 17AAG, at two different doses, and the cytotoxic effect was evaluated by a trypan blue assay after 24 h of treatment. As shown in Figure 1B, all three HSP inhibitors impaired PEL cell survival in a dose-dependent fashion. As a control, the cytotoxicity of all HSP inhibitors was evaluated against primary B lymphocytes and, as shown in Supplementary Figure S1, they slightly influenced B-cell survival. To evaluate the mechanisms leading to cell death following the treatment with these inhibitors, caspase-3 cleavage was evaluated by Western blot analysis. We found that HSP27 and HSP90, but not HSP70, inhibition triggered caspase-3 cleavage (ClCasp3), inducing apoptotic cell death (Figure 1C). Regarding HSP70 inhibitors, according to our previous findings [7], we confirmed that this treatment resulted in a necroptotic cell death in PEL cells, based on the appearance of annexin V/PI double-positive cells, after 6 h of treatment (Figure 1D).

### 2.2. HSP Inhibition Induces DNA Damage in PEL Cells, Which Correlates with the Downregulation of Molecules Involved in DNA Repair

**Figure 2:**
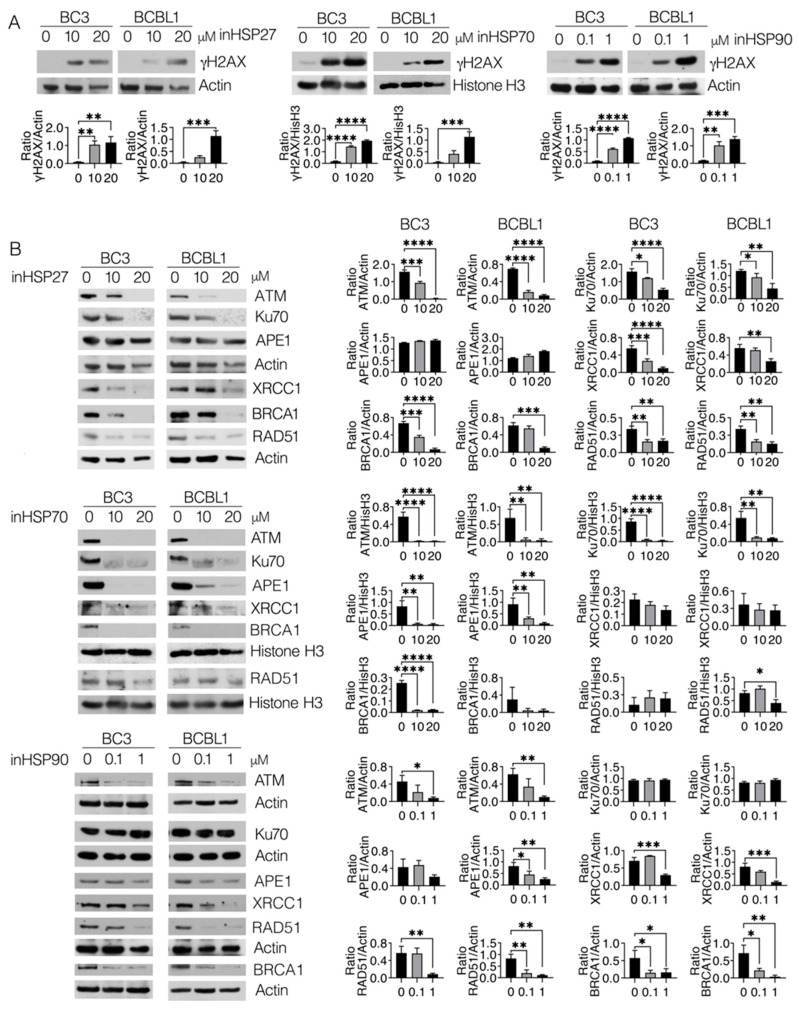
HSP inhibition induces DNA damage and DDR molecule downregulation in PEL cells. BC3 and BCBL1 cells were treated with inHSP27 (J2) (10–20 µM), inHSP70 (PES) (10–20 µM) or inHSP90 (17AAG) (0.1–1 µM) for 24 h and (**A**) protein expression levels of γH2AX were evaluated by Western blot analysis. Actin or histone H3 was used as a loading control and one representative experiment is shown. The histograms represent the densitometric analysis of the ratio of γH2AX/actin or histone H3. Data are represented as the mean plus S.D. *p* value: ** <0.01; *** <0.001; **** <0.0001. (**B**) Protein expression levels of ATM, Ku70, APE1, XRCC1, BRCA1 and RAD51 were evaluated by Western blot analysis. Actin or histone H3 was used as a loading control and one representative experiment is shown. The histograms represent the densitometric analysis of the ratio of specific protein/actin or histone H3. Data are represented as the mean plus S.D. *p* value: * <0.05; ** <0.01; *** <0.001; **** <0.0001.

As HSPs may sustain the expression levels of molecules essential for repairing DNA damage [16], we evaluated the impact of HSP inhibition on DNA damage in PEL cells. As shown in Figure 2A, HSP27, 70 and 90 inhibitors increased the expression levels of γH2AX in a dose-dependent fashion, which suggests the occurrence of DNA damage. We then explored whether such effects could correlate with a reduced expression of molecules involved in major DNA damage repair pathways. We found that HSP27 inhibitor (J2) reduced the expression level of ATM, a kinase activating the DNA repair signaling cascade in response to DNA double-strand breaks (Figure 2B). Ku70, belonging to NHEJ, XRCC1, but not APE1, involved in BER, BRCA1 and RAD51 of the HR pathway, were also downregulated by HSP27 inhibition. The expression level of all the above-mentioned molecules, except for RAD51 that was slightly affected, was also reduced by HSP70 inhibitor (PES) (Figure 2B). Moreover, this treatment downregulated APE1, a molecule not affected by HSP27 targeting (Figure 2B). The HSP90 inhibitor (17AAG) downregulated ATM, XRCC1, BRCA1 and RAD51, while not influencing the expression of Ku70 and slightly affecting APE1 (Figure 2B). However, HSP90 inhibition could activate HSF1, as a compensatory mechanism. Indeed, here we found that treatment with 17AAG upregulated HSP27 and, to a smaller extent, HSP70, an effect that could reduce the efficacy of HSP90 inhibition (Supplementary Figure S2A). Moreover, the concomitant inhibition of HSP27, HSP70 and HSP90, used at the lowest doses, induced a dramatic reduction of PEL cell survival (Supplementary Figure S2B). From this study, it emerges that the stability of many DDR molecules depends on the presence of several HSPs. Some molecules have been previously reported to be stabilized by these HSPs [16], while for others, we obtained different results in terms of dependency on HSP27, HSP70 or HSP90.

### 2.3. Targeting HSP27, HSP70 and HSP90 Inhibits STAT3 and Cytokine Release by PEL Cells

**Figure 3:**
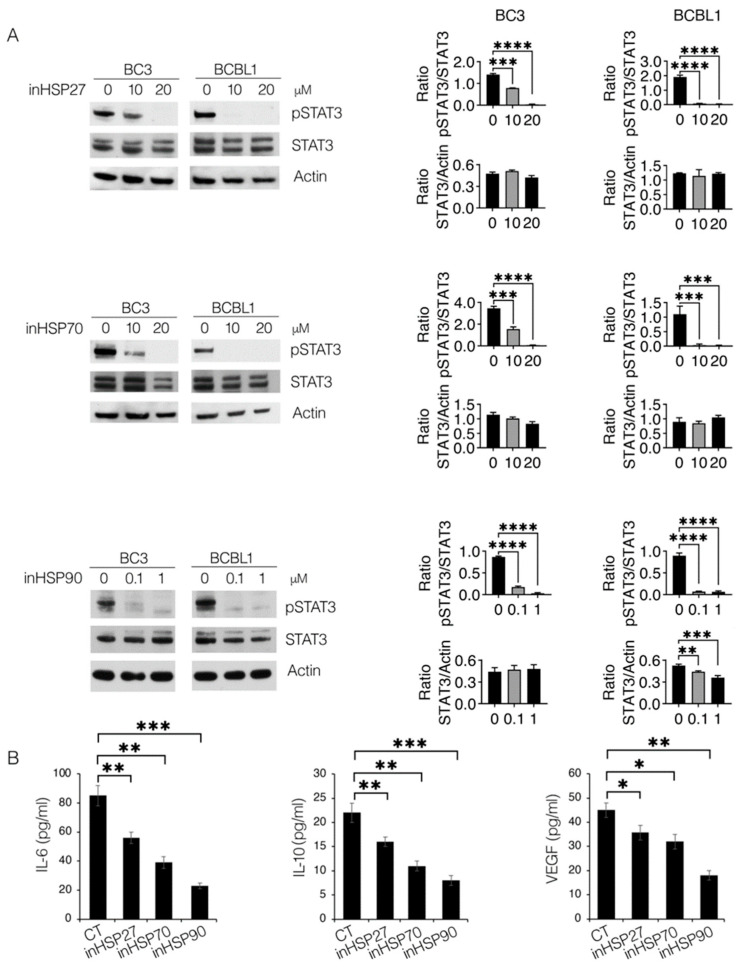
HSP inhibition reduces STAT3 activation and cytokine release in PEL cells. BC3 and BCBL1 cells were treated with inHSP27 (J2) (10–20 µM), inHSP70 (PES) (10–20 µM) or inHSP90 (17AAG) (0.1–1 µM) for 24 h and (**A**) protein phosphorylation of STAT3 was evaluated by Western blot analysis. Actin was used as a loading control and one representative experiment is shown. The histograms represent the densitometric analysis of the ratio of specific protein/actin. Data are represented as the mean plus S.D. *p* value: ** <0.01; *** <0.001; **** <0.0001. (**B**) After treatment with inHSP27 (10 µM), inHSP70 (10 µM) or inHSP90 (0.1 µM), supernatants from BC3 were collected and analyzed for IL-6, IL-10 and VEGF amount by a Luminex Assay. Histograms representing the mean ± S.D. of the three independent experiments. *p*-value: * <0.05; ** <0.01; *** <0.001.

Among other numerous functions, HSPs have been reported to chaperone kinases that sustain pathways such as STAT3 [18]. It is constitutively phosphorylated in PEL and plays an important role in its survival [1]. In this study, the impact of HSP inhibitors on STAT3 activation was investigated in these cells. As shown in Figure 3A, STAT3 phosphorylation was reduced by treatment with inHSP27 (J2), inHSP70 (PES) and inHSP90 (17AAG), in both BC3 and BCBL1 cells. As this transcription factor is strongly interconnected with the release of cytokines, such as IL-6, IL-10 and VEGF [19,20], that contribute to PEL cells growth [5], we performed a Luminex assay to evaluate their production by PEL cells undergoing treatment with HSP27, HSP70 and HSP90 inhibitors. Although the HSP90 inhibitor was more effective, all three HSP inhibitors impaired the release of these cytokines (Figure 3B), an effect that could contribute to their cytotoxic effects against PEL.

### 2.4. STAT3 Inhibition Downregulates HSP27, HSP70 and HSP90, as Well as DDR Molecules in PEL Cells

**Figure 4:**
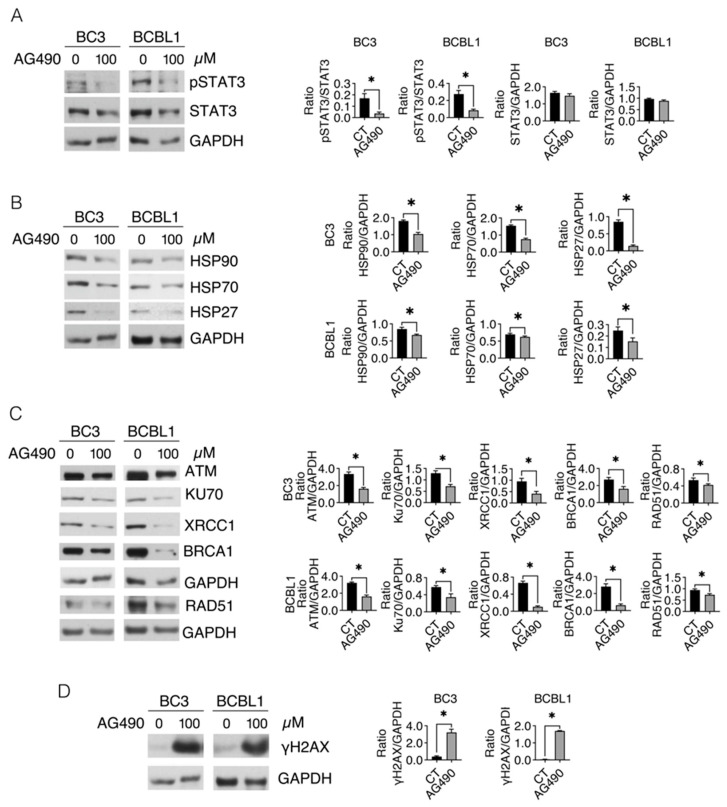
STAT3 inhibition downregulates HSPs and DDR expression and induces DNA damage in PEL cells. BC3 and BCBL1 cell lines were treated with AG490 (100 µM) for 24 h and (**A**) protein phosphorylation of STAT3 was evaluated by Western blot analysis. GAPDH was used as a loading control and one representative experiment is shown. The histograms represent the densitometric analysis of the ratio of specific protein/GAPDH. Data are represented as the mean plus S.D. *p* value: * <0.05. (**B**) Protein expression levels of HSP27, HSP70 and HSP90 were evaluated by Western blot analysis. GAPDH was used as a loading control and one representative experiment is shown. The histograms represent the densitometric analysis of the ratio of specific protein/GAPDH. Data are represented as the mean plus S.D. *p* value: * <0.05. (**C**) Protein expression levels of ATM, XRCC1, Ku70, BRCA1 and RAD51 were evaluated by Western blot analysis. GAPDH was used as a loading control and one representative experiment is shown. The histograms represent the densitometric analysis of the ratio of specific protein/GAPDH. Data are represented as the mean plus S.D. *p* value: * <0.05. (**D**) Protein expression levels of γH2AX were evaluated by Western blot analysis. GAPDH was used as a loading control and one representative experiment is shown. The histograms represent the densitometric analysis of the ratio of γH2AX/GAPDH. Data are represented as the mean plus S.D. *p* value: * <0.05.

STAT3 activation, which may be sustained by HSPs, has been shown to contribute to their transcription [4,13,18]. Accordingly, here we found that AG490, by inhibiting STAT3 (Figure 4A), reduced the expression of HSP27, HSP70 and HSP90, in both PEL cell lines (Figure 4B). Based on the interconnections between STAT3 and HSPs, and HSPs and DDR, we then explored whether STAT3 inhibition could influence the expression of DDR molecules in PEL cells. As shown in Figure 4C, AG490 reduced ATM and more efficiently downregulated Ku70, XRCC1, BRCA-1 and RAD51. Accordingly, STAT3 inhibition increased DNA damage, as indicated by the increased expression of γH2AX (Figure 4D), suggesting that the positive feedback loop between HSPs and STAT3 plays a key role in sustaining DDR and preventing DNA damage in PEL cells.

### 2.5. HSP, as Well as STAT3 Inhibition, May Sensitize PEL Cells to the Cytotoxic Effect of PARP Inhibitor

**Figure 5:**
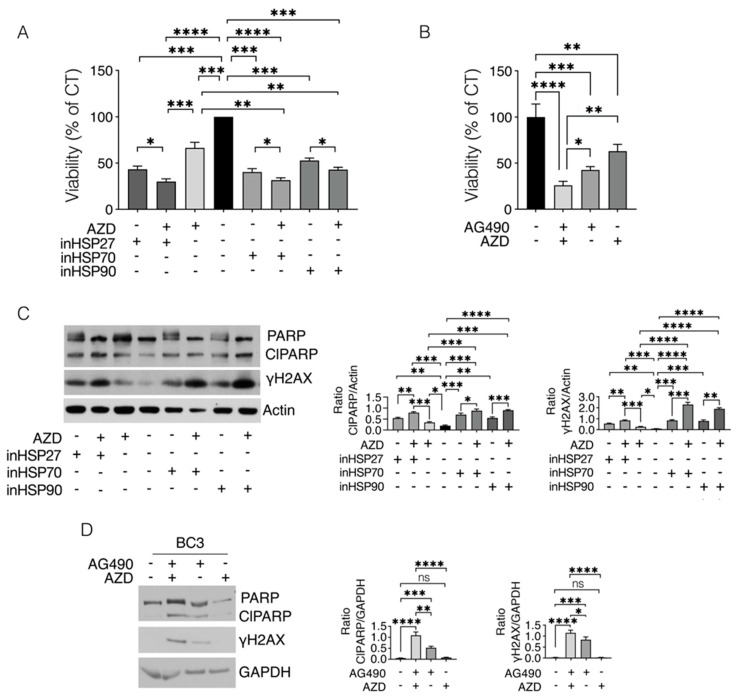
HSP or STAT3 inhibitors potentiate the cytotoxic effect of PARP inhibitor AZD2461 in PEL cells, by inducing stronger DNA damage. BC3 cell lines were treated with inHSP27 (J2) (10 µM), inHSP70 (PES) (10 µM), inHSP90 (17AAG) (0.1 µM) or AG490 (100 µM) singly or in combinations with AZD2461 (40 μM) for 24 h and (**A**,**B**) cell survival was evaluated by a trypan blue exclusion assay. The histograms represent the mean of the percentage of cell viability relative to the control plus S.D. *p* value: * <0.05; ** <0.01; *** <0.001; **** <0.0001. By calculating the KERN index, the cytotoxic effect, induced by all combinations, was found to be additive (R = 1). (**C**,**D**) Protein expression levels of cleaved PARP (ClPARP) and γH2AX were evaluated by Western blot analysis. Actin or GAPDH was used as a loading control and one representative experiment is shown. The histograms represent the densitometric analysis of the ratio of specific protein/actin or protein/GAPDH. Data are represented as the mean plus S.D. *p* value: * <0.05; ** <0.01; *** <0.001; **** <0.0001. ns = not significant.

As HSPs and STAT3 influence each other and sustain DDR, we then investigated whether their inhibition could sensitize PEL cells to the cytotoxic effect of PARP inhibitor, AZD2461 (AZD), previously shown to be effective in reducing PEL survival [21]. As shown in Figure 5A,B, treatment with HSP27, HSP70 and HSP90 inhibitors (J2, PES and 17AAG, respectively), as well as by AG490 rendered PEL cells more susceptible to AZD treatment, as evaluated by a trypan blue assay. Moreover, a stronger PARP cleavage (ClPARP) and an increase in γH2AX was observed following treatment with HSP or STAT3 inhibitors in combinations with AZD, compared to the single treatments (Figure 5C,D), which also suggests the occurrence of stronger apoptotic cell death and DNA damage. All together, these results suggest that HSP or STAT3 inhibition could be exploited to potentiate the cytotoxic effect of PARP inhibitor and possibly of other DNA damaging against PEL.

## 3. Discussion

HSPs play a key role in the stabilization and function of proteins involved in important biological processes, therefore targeting HSPs may have a strong impact on cell survival [14]. Their role is particularly important in cancer cells, as they express high levels of HSPs and strongly rely on them for protein-folding and for adapting to the stressful conditions that characterize their life [8]. This is also due to the fact that HSPs contribute to the stabilization of oncogenes, such as c-MYC [22] and mutant KRAS [23]. Previous studies from other laboratories, along with ours, have shown that targeting HSP70, as well as HSP90, could represent a successful strategy to kill PEL cells by necroptotic or apoptotic cell death, respectively [7,12].

In this study, we confirmed the efficacy of HSP70 and HSP90 inhibition as a promising therapeutic option against this aggressive lymphoma associated to the oncovirus KSHV, and unveiled new molecular mechanisms leading to the cytotoxicity induced by these HSP inhibitors. Indeed, we found that they caused strong DNA damage and downregulated several molecules involved in important DNA repair pathways in PEL cells. Here, we also show for the first time that HSP27 inhibition can be a very effective strategy against PEL cells, as this treatment also induced DNA damage and reduced the expression levels of several DDR molecules, similarly to HSP70 and HS90 inhibition. Some DDR molecules have been previously shown to be stabilized by HSP70 and HSP90 in other cell types [16], while others in this study were found to either be or not be clients of HSP27, HSP70 or HSP90, proteins that play a leading role in the safeguard of genome integrity. Finding strategies that impair DDR is particularly important, not only because this response is required to repair DNA damage that frequently occurs in basal conditions in cancer cells, but also because cancer cells characterized by “BRCAness”, those carrying mutations in the genes encoding DDR molecules, are more susceptible to anti-cancer treatments by DNA-damaging agents or inhibitors of PARP, as well as other molecules involved in DNA repair [24]. However, “BRCAness” can be pharmacologically induced in cancer cells that do not harbor mutations in DDR genes, as suggested, for example, by previous studies, in which we used HDAC inhibitors to impair DDR molecules in pancreatic cancer cells [25,26].

As another important molecular mechanism leading to PEL cell death, here we found that targeting HSP27, HSP70 and HSP90 inhibited STAT3. Accordingly, among other proteins, it has been reported that HSPs can stabilize kinases able to phosphorylate STAT3, such as JAK2 [18]. The finding that HSP inhibitors reduced STAT3 activation is important not only because STAT3 promotes the transcription of pro-survival molecules, such as survivin, cyclin D1 and c-MYC [27], but also because STAT3 promotes the release of cytokines, including IL-6, IL-10 and VEGF. These are known to support PEL cell growth [5] and importantly, they may also contribute to the cancer-induced immune dysfunction [28]. Therefore, targeting HSPs could allow to restore anti-cancer immune response, by interrupting the interplay between STAT3 and cytokine production. The cross-talk between STAT3 and HSPs found in this study is in agreement with previous reports, showing that the inhibition of STAT3 could lead to DNA damage [29] or downregulate HSPs, including HSP90 and HSP27 [4,13,30].

Interestingly, as this study suggests, it is emerging that interrupting the positive feedback loop between HSPs and STAT3 could interfere with a variety of pro-survival processes, not only inside, but also outside the cells, which is quite a new aspect that may have important implications in the treatment of cancer.

## 4. Materials and Methods

### 4.1. Cell Cultures and Treatments

KSHV-positive PEL cell lines, BC3 (ATCC, CRL-2277) and BCBL1 (kindly supplied by Prof. P. Monini, National AIDS center, Istituto Superiore di Sanità, Rome, Italy), were grown in RPMI 1640 medium (Sigma-Aldrich, Burlington, MA, USA), supplemented with 10% fetal bovine serum (FBS) (Sigma-Aldrich, Burlington, MA, USA), L-glutamine (2 mM) (Aurogene, Rome, Italy), and streptomycin/penicillin (100 μg/mL) (Aurogene, Rome, Italy) (complete medium) at 37 °C, in a 5% CO_2_ humified setting. Cells were seeded into 6-well plates, at a density of 4 × 10^5^ per well, in a final volume of 2 mL in complete RPMI medium, and they were treated for 24 h with J2 (10–20 µM) (MedChemExpress, Monmouth Junction, NJ, USA; cat n. HY-124653), PES-Cl (10–20 µM) (Calbiochem, San Diego, CA, USA; cat n. 5.31067), 17-AAG (0.1–1 µM) (Selleckem, Planegg, Germany; cat. n. S1141) or AG490 (100 µM) (Calbiochem, San Diego, CA, USA; cat n. 658411), inhibitors of HSP27, HSP70, HSP90 and STAT3, respectively. All the drugs were dissolved in DMSO, and the control cells were supplemented with DMSO in the same amount used for the other samples. To evaluate if HSPs or STAT3 inhibitors could sensitize PEL cells to the cytotoxic effect of PARP inhibitor, the cell lines were pre-treated for 1 h with HSP27, 70, 90 inhibitor (inHSP) or AG490 and then supplemented with AZD2461 (AZD, 40 μM) (Sigma-Aldrich, Burlington, MA, USA, cat n. SML1858) for 24 h. Untreated cells were used as a control group (CT).

### 4.2. CD19 B-Cell Isolation

Human peripheral blood mononuclear cells (PBMC) were separated by Ficoll-Paque gradient centrifugation (Lympholyte; Cedarlane, CL5020) from buffy coats of healthy donors. B lymphocytes were isolated from PBMCs by immunomagnetic cell separation, using anti-CD19-conjugated microbeads, according to the manufacturer’s instructions (Miltenyi Biotec, 130–050-301), and were plated in 6-well plates at a density of density of 4 × 10^6^ per well, in a final volume of 2 mL in MC, and treated for 24 h with J2 (20 µM), PES (20 µM) and 17-AAG (1 µM), inhibitors of HSP27, HSP70 and HSP90, respectively.

### 4.3. Trypan Blue Exclusion Assay

The trypan blue dye exclusion assay was used to determine the number of viable cells. Following treatments, the cell suspension was mixed with trypan blue (Sigma-Aldrich, Burlington, MA, USA; cat. n. T8154) and live cells, that exclude the dyes, were counted by light microscopy, using a Neubauer hemocytometer. The experiments were performed in triplicate and repeated at least three times.

### 4.4. Annexin/PI Staining

After inHSP treatments, the cells were washed with ice-cold PBS, resuspended in annexin V binding buffer, and subsequently stained with annexin V–fluorescein isothiocyanate (FITC) and PI (BD Pharmingen, San Jose, CA, USA; cat n. 556547), according to the manufacturer’s recommendation. The cells were analyzed by FACSCalibur (BD Biosciences, East Rutherford, NJ, USA). Data are representative of at least three independent experiments and analyzed by CellQuest (BD Biosciences, East Rutherford, NJ, USA). Debris and dead cells were excluded from the analysis, gating live cells in a forward versus side scatter (FSC vs. SSC) density plot. For each analysis, 10,000 events were recorded.

### 4.5. Western Blot Analysis

After treatments, cells were harvested, centrifuged at 1200 rpm (revolutions per minute) for 5 min at room temperature (RT) and lysed in the RIPA buffer (150 mM NaCl, 1% NP-40, 50 mM Tris-HCl (pH 8), 0.5% deoxycholic acid, 0.1% SDS, protease and phosphatase inhibitors). Protein concentration was measured by using a quick-start bovine serum albumin (BSA) assay (Bio-Rad, Hercules, CA, USA; cat n. 5000206), and 10 μg of proteins of each sample were denatured in loading buffer by heating for 10 min at 70 °C, and subsequently subjected to electrophoresis on 4–12% NuPage Bis-Tris gels (Life Technologies, Carlsbad, CA, USA), according to the manufacturer’s instruction. Proteins were then transferred to nitrocellulose membranes (Amersham, Buckinghamshire, UK, cat n. 10600002) for 45 min in the tris-glycine buffer [31]. Afterwards, membranes were blocked in 1x PBS-0.1% Tween 20 solution, containing 3% of BSA (Applichem, GMBH, Darmstadt, Germany cat n. A1391,0100) for 1 h at RT, then incubated with specific primary antibodies over night at 4 °C. The day after, the blots were washed three times with 1x PBS-0.1% Tween 20 and incubated with the proper secondary antibody HRP, conjugated at a dilution of 1:10,000 for 30 min at room temperature. After three washes with PBS-0.1% Tween 20, the blots were finally subjects to ECL (Advansta, San Jose, CA, USA, cat n. 12045-D20).

### 4.6. Antibodies

To identify specific proteins in western blot analyses the following primary antibodies were used: rabbit polyclonal anti-PARP (1:1000) (Cell Signaling, Danvers, MA, USA, cat n. 9542), rabbit polyclonal anti-phospho STAT3 Tyr705 (1:500) (Cell Signalling Danvers, MA, USA, cat n. 9145), mouse monoclonal anti-STAT3 (1:500) (Santa Cruz Biotechnology Inc, Dallas, TX, USA, cat n. sc-482), mouse monoclonal anti-APE (Ref-1) (1:500) (Santa Cruz Biotechnology Inc, Dallas, TX, USA, cat n. sc-17774), mouse monoclonal anti-ATM (1:200) (Santa Cruz Biotechnology Inc, Dallas, TX, USA, cat n. sc-135663), mouse monoclonal anti-BRCA-1 (1:1000) (EMD Millipore, Burlington, MA, cat n. OP92), mouse monoclonal anti-caspase-3 (1:500) (Santa Cruz Biotechnology Inc., Dallas, TX, USA, cat n. sc-56053), mouse monoclonal anti-pH2AX (Ser 139) (1:500) (Santa Cruz Biotechnology Inc., Dallas, TX, USA, cat n. sc-517348), mouse monoclonal anti-HSP27 (1:1000) (Proteintech, Rosemont, IL, USA, cat n. 182841), mouse monoclonal anti-HSP70 (1:500) (Santa Cruz Biotechnology Inc, Dallas, TX, USA, cat n. sc-32239), mouse monoclonal anti-HSP90 (1:1000) (Santa Cruz Biotechnology Inc, Dallas, TX, USA, cat n. sc-69703), mouse monoclonal anti-KU70 (1:200) (Santa Cruz Biotechnology Inc., Dallas, TX, USA, cat n. sc-17789), mouse monoclonal anti-RAD51 (1:200) (Santa Cruz Biotechnology Inc, Dallas, TX, USA, cat n. sc-377467), mouse monoclonal anti-XRCC1 (1:100) (Santa Cruz Biotechnology Inc., Dallas, TX, USA, cat n. sc-56254). The following antibodies were used as loading controls secondary antibodies: mouse monoclonal anti-β-Actin (1:10,000) (Sigma Aldrich, St. Louis, MO, USA cat n. A5441), mouse monoclonal anti-GAPDH (1:10,000) (Santa Cruz Biotechnology Inc., Dallas, TX, USA, cat n. 47724), rabbit polyclonal anti-Histone H3 (1:500) (Cell Signalling Danvers, MA, USA, cat n. 9715). The goat anti-mouse IgGP peroxidase conjugate (Sigma-Aldrich, 401215, Burlington, MA, USA) and the goat anti-rabbit IgG peroxidase conjugate (DC03L; Sigma-Aldrich, Burlington, MA, USA).All the primary and secondary antibodies were diluted in 1x PBS-0.1% Tween 20 solution, containing 2% of BSA.

### 4.7. Chemiluminescent Immunometric Assay (Luminex Assay)

After HSP inhibition, as reported above, supernatants from BC3 were collected and interleukin-6 (IL-6), interleukin-10 (IL-10) and vascular endothelial growth factor (VEGF) were measured by a magnetic Luminex assay, using a human pre-mixed multi-analyte kit (R&D systems Bio-Techne, LXSAHM), according to the manufacturer’s instructions.

### 4.8. Densitometric Analysis

Densitometric analysis on Western blots were performed by using the Image J software (1.47 version, NIH, Bethesda, MD, USA), which was downloaded from the NIH website (http://imagej.nih.gov (accessed on 10 February 2022)).

### 4.9. Statistical Analysis

Results are represented by the mean plus standard deviation (S.D.) of at least three independent experiments and statistical analyses were performed with the Graphpad Prism^®^ software (Graphpad software Inc., La Jolla, CA, USA). A student’s t test or a nonparametric one-way ANOVA test were used to demonstrate statistical significance. Difference was considered statistically significant when the *p*-value was: * <0.05; ** <0.01; *** <0.001 and **** <0.0001.

## Data Availability

The datasets generated and analyzed during the current study are available from the corresponding author upon reasonable request.

## Supplementary Materials

The following supporting information can be downloaded at: https://www.mdpi.com/article/10.3390/ijms24043933/s1.
