# Supplementary material for: HSPs/STAT3 Interplay Sustains DDR and Promotes Cytokine Release by Primary Effusion Lymphoma Cells
Source: Int J Mol Sci. 2023 Feb 15;24(4):3933. doi: 10.3390/ijms24043933 (PMC9959463; doi:10.3390/ijms24043933)
Supplement: Supplementary file 1 [file ijms-24-03933-s001.zip › ijms-2210341-supplementary.pdf]

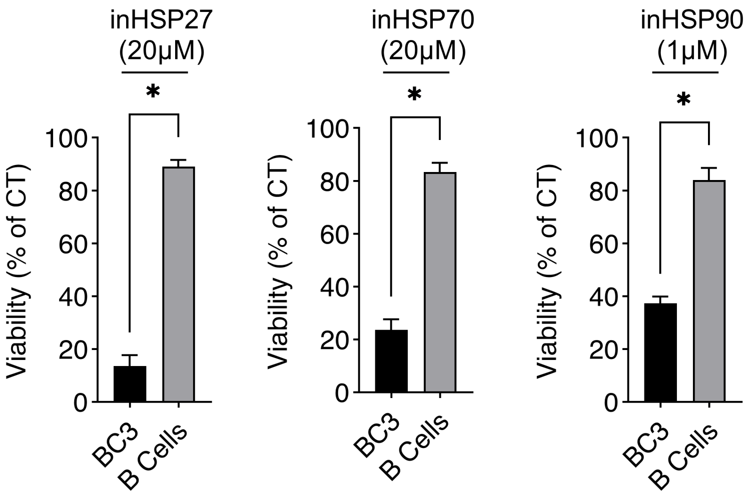

inHSP27
(20µM)
*
Viability (% of CT)
100
80
60
40
20
0
BC3
B Cells
inHSP70
(20µM)
*
Viability (% of CT)
100
80
60
40
20
0
BC3
B Cells
inHSP90
(1µM)
*
Viability (% of CT)
100
80
60
40
20
0
BC3
B Cells

Figure S1

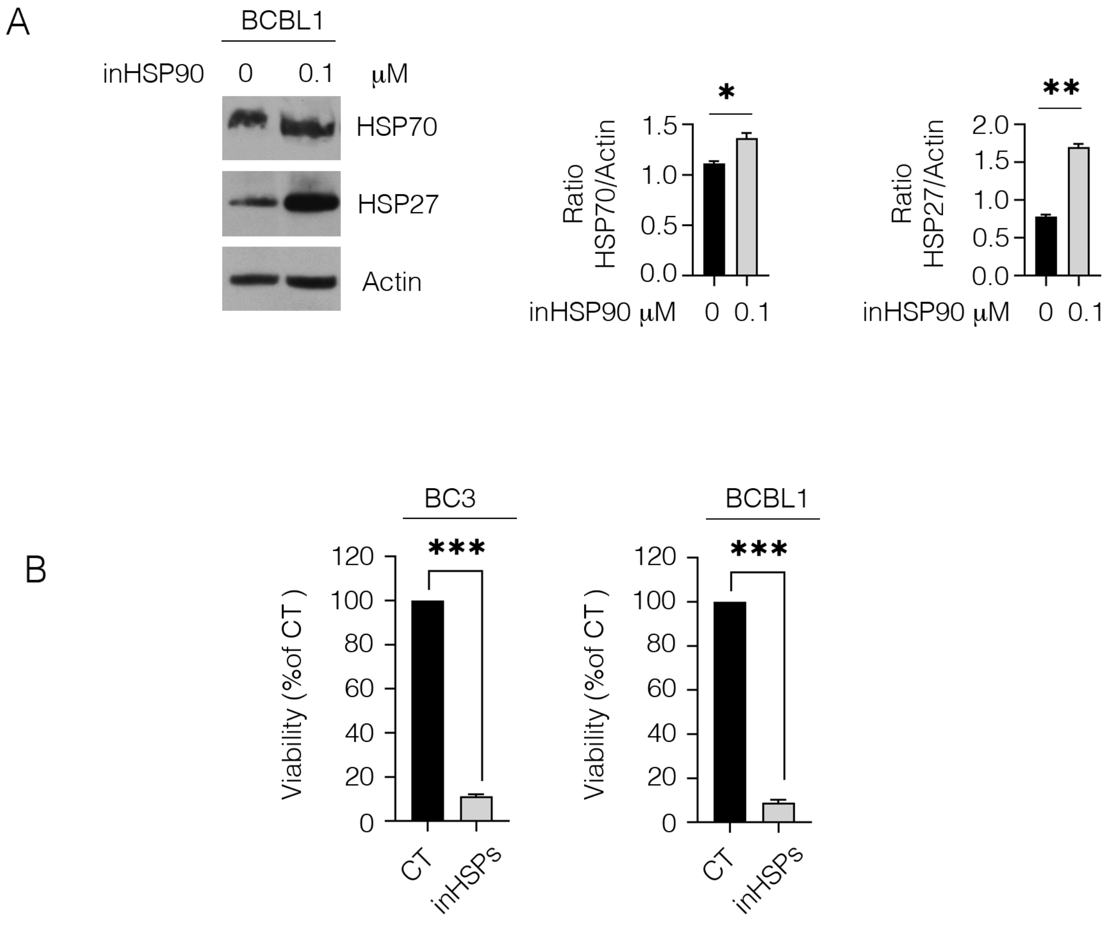
A
BCBL1
inHSP90 0 0.1 μM
HSP70
HSP27
Actin
*
Ratio HSP70/Actin
1.5
1.0
0.5
0.0
inHSP90 μM 0 0.1
**
Ratio HSP27/Actin
2.0
1.5
1.0
0.5
0.0
inHSP90 μM 0 0.1
B
BC3
***
Viability (%of CT)
120
100
80
60
40
20
0
CT
inHSPs
BCBL1
***
Viability (%of CT)
120
100
80
60
40
20
0
CT
inHSPs

Figure S2

Supplementary figure legends

Fig.S1 Comparison between the toxicity exerted by HSP27 inhibitor (J2) (10μM), HSP70 inhibitor (PES) (10μM) and HSP90 inhibitor (17AAG) (0.1μM) against PEL cells and primary B lymphocytes, as evaluated by trypan blue assay. The histograms represent the mean of percentage of cell viability relative to the control plus S.D. p value: * <0.05; **<0.01; ***<0.001

Fig.S2 A) Effect of HSP90 inhibitor (17AAG) (0.1μM) on HSP70 and HSP27 expression level in PEL cells, as evaluated by western blot analysis. The histograms represent the densitometric analysis of the ratio of specific protein/Actin. Data are represented as the mean plus S.D. p value: * <0.05; **<0.01.

B) Cytotoxic effect of HSP27, 70 and 90 inhibition on PEL cells as evaluated by trypan blue assay. The histograms represent the mean of percentage of cell viability relative to the control plus S.D. p value: * <0.05; **<0.01; ***<0.001.
